# Interpreting Prefrontal Recruitment During Walking After Stroke: Influence of Individual Differences in Mobility and Cognitive Function

**DOI:** 10.3389/fnhum.2019.00194

**Published:** 2019-06-18

**Authors:** Sudeshna A. Chatterjee, Emily J. Fox, Janis J. Daly, Dorian K. Rose, Samuel S. Wu, Evangelos A. Christou, Kelly A. Hawkins, Dana M. Otzel, Katie A. Butera, Jared W. Skinner, David J. Clark

**Affiliations:** ^1^Brain Rehabilitation Research Center (BRRC), Malcom Randall VA Medical Center, Gainesville, FL, United States; ^2^Department of Physical Therapy, University of Florida, Gainesville, FL, United States; ^3^Brooks Rehabilitation, Jacksonville, FL, United States; ^4^Department of Neurology, University of Florida, Gainesville, FL, United States; ^5^Department of Biostatistics, University of Florida, Gainesville, FL, United States; ^6^Department of Applied Physiology and Kinesiology, University of Florida, Gainesville, FL, United States; ^7^Department of Aging and Geriatric Research, University of Florida, Gainesville, FL, United States; ^8^Geriatric Research, Education and Clinical Center, Malcom Randall VA Medical Center, Gainesville, FL, United States

**Keywords:** dual-task walking, near-infrared spectroscopy, prefrontal cortex, stroke, walking

## Abstract

**Background**: Functional near-infrared spectroscopy (fNIRS) is a valuable neuroimaging approach for studying cortical contributions to walking function. Recruitment of prefrontal cortex during walking has been a particular area of focus in the literature. The present study investigated whether task-related change in prefrontal recruitment measured by fNIRS is affected by individual differences in people post-stroke. The primary hypotheses were that poor mobility function would contribute to prefrontal over-recruitment during typical walking, and that poor cognitive function would contribute to a ceiling in prefrontal recruitment during dual-task walking (i.e., walking with a cognitive task).

**Methods**: Thirty-three adults with chronic post-stroke hemiparesis performed three tasks: typical walking at preferred speed (*Walk*), serial-7 subtraction (*Serial7*), and walking combined with serial-7 subtraction (*Dual-Task*). Prefrontal recruitment was measured with fNIRS and quantified as the change in oxygenated hemoglobin concentration (ΔO_2_Hb) between resting and active periods for each task. Spatiotemporal gait parameters were measured on an electronic walkway. Stepwise regression was used to assess how prefrontal recruitment was affected by individual differences including age, sex, stroke region, injured hemisphere, stroke chronicity, 10-meter walking speed, balance confidence measured by Activities-specific Balance Confidence (ABC) Scale, sensorimotor impairment measured by Fugl-Meyer Assessment, and cognitive function measured by Mini-Mental State Examination (MMSE).

**Results**: For *Walk*, poor balance confidence (ABC Scale score) significantly predicted greater prefrontal recruitment (ΔO_2_Hb; *R*^2^ = 0.25, *p* = 0.003). For *Dual-Task*, poor cognitive function (MMSE score) significantly predicted lower prefrontal recruitment (ΔO_2_Hb; *R*^2^ = 0.25, *p* = 0.002).

**Conclusions**: Poor mobility function predicted higher prefrontal recruitment during typical walking, consistent with compensatory over-recruitment. Poor cognitive function predicted lower prefrontal recruitment during dual-task walking, consistent with a recruitment ceiling effect. These findings indicate that interpretation of prefrontal recruitment should carefully consider the characteristics of the person and demands of the task.

## Introduction

Functional near-infrared spectroscopy (fNIRS) has emerged as a valuable measure for assessing the cortical contributions to locomotor control in both healthy and neurologically impaired populations (Mihara et al., 2007; Holtzer et al., 2011; Clark et al., 2014; Lu et al., 2015; Al-Yahya et al., 2016; Maidan et al., 2016b; Chen et al., 2017; Mirelman et al., 2017; Hawkins et al., 2018; Herold et al., 2018; Mori et al., 2018). The prefrontal cortex is an important region that has been examined extensively with fNIRS during walking. Task-related recruitment of prefrontal cortex infers a demand for cognitive/executive control resources, which support attention, working memory, motor planning, and task switching (Yogev-Seligmann et al., 2008; Al-Yahya et al., 2009; Amboni et al., 2013). These cognitive domains play an important role during every day walking in the home and community, especially in challenging environments or under distracted conditions (Suzuki et al., 2004; Yogev-Seligmann et al., 2008; Caliandro et al., 2012; Amboni et al., 2013).

An important challenge for studies investigating brain activation during walking is that increased recruitment could be interpreted as either an appropriate or an atypical neural control strategy. Therefore, it is important to carefully consider the demands of the task(s) as well as the characteristics of the study participants when interpreting brain recruitment.

### When Is Higher Prefrontal Recruitment Appropriate?

Greater brain recruitment is beneficial when it enables the individual to meet the demands of high complexity tasks (Cabeza, 2002; Cabeza et al., 2002, 2004; Reuter-Lorenz and Cappell, 2008). In this context, higher recruitment of prefrontal cortex conveys robust availability and utilization of executive resources. In contrast, deficient cognitive function can impose a lower “ceiling” of brain resource recruitment and thereby limit performance on tasks that require those resources (Reuter-Lorenz and Cappell, 2008). For instance, Cabeza et al. (2002) reported that compared to lower performing older adults, higher performing older adults exhibited greater prefrontal recruitment during cognitive tasks (recall and source memory of words) and better task performance. They posited that higher performing older adults were able to counter age-related decline in neural networks by recruiting bilateral neurocognitive networks. Likewise, Reuter-Lorenz et al. (2000) have also reported that older adults who exhibited greater prefrontal recruitment (i.e., bilateral recruitment) also exhibited faster performance on a verbal working memory task. In agreement, similar findings have also been reported in the context of walking. Compared to older adults, younger adults exhibit a more effective utilization of prefrontal resources (achieved by higher recruitment) and better task performance during dual-task walking (Holtzer et al., 2011). This finding may be consistent with reports of age-related atrophy in the sensorimotor and frontoparietal areas (Rosano et al., 2008) and associated decline in attention, psychomotor processing speed and problem solving, and increased fall risk during walking in the elderly population (Gauchard et al., 2006; Alexander and Hausdorff, 2008; Herman et al., 2010; Fasano et al., 2012; Liu et al., 2014). Furthermore, older adults who exhibit a greater increase in prefrontal recruitment during complex walking tasks demonstrate better task performance, including a smaller reduction in walking speed and lower step length variability relative to typical walking (Clark et al., 2014). Likewise, increased recruitment in the frontal cortex is associated with improved walking performance (i.e., reduced gait variability) in older adults following rhythmic auditory cueing during walking (Vitorio et al., 2018).

### When Is Higher Prefrontal Recruitment Atypical?

It is important to consider that major contributors to neural control of walking reside at lower levels of the neuraxis. These include brainstem regions, spinal pattern generating circuits, and cerebellar circuits that use both descending and afferent (e.g., somatosensory) inputs to generate patterns of intermuscular and interlimb coordination (Nielsen, 2003; Grillner, 2011). These mechanisms promote automaticity of walking, such that demand for executive control resources is minimized (Clark, 2015). Any impairment that disrupts these circuits of automaticity may give rise to compensatory recruitment of prefrontal resources to augment control (Clark et al., 2014; Maidan et al., 2016b; Hawkins et al., 2018).

Compensatory prefrontal recruitment for control of walking might be further exacerbated by impairments within the brain. Prior investigations from the cognitive literature suggest that neural mechanisms contributing to brain over-recruitment include: (1) inefficient processing, such that greater amounts of brain recruitment are required to achieve a given level of task performance; (2) poor specificity of recruiting specialized networks, thereby leading to widespread recruitment; (3) reactive recruitment to poor task performance in an attempt to improve performance; and (4) compensatory recruitment that is elicited proactively to support task performance when primary brain regions/network recruitment is deficient (Cabeza et al., 2004, 2002; Cabeza, 2002; Reuter-Lorenz and Cappell, 2008; Goh and Park, 2009). Regardless of the specific causes of prefrontal over-recruitment, this phenomenon encumbers resources and hastens reaching the recruitment ceiling, particularly under conditions of increased task complexity (Reuter-Lorenz and Cappell, 2008). Since walking is a complex task that utilizes cognitive resources, the aforementioned cognitive control scenarios of over-recruitment and ceiling effect might also influence walking function.

### Prefrontal Recruitment During Walking in Adults Post-stroke

Stroke is a leading cause of long-term physical disability, with devastating consequences that often include loss of independence, restricted participation in life roles, and a decline in quality of life (Lord et al., 2004; D’Alisa et al., 2005; Rosen et al., 2005; Robinson et al., 2011; Schmid et al., 2012). Since the ability to walk safely in the community is critical for preservation of independence, social integration, and participation in life-roles, recovery of walking function is often the most emphasized rehabilitation goal after stroke (Bohannon et al., 1988; Lord et al., 2004; Pang et al., 2007). Stronger improvements in rehabilitation outcomes might be possible if mechanistic targets can be more accurately identified and understood. One such target is the high demand for executive/prefrontal control resources during walking in adults post-stroke (Hawkins et al., 2018).

The objective of this study was to investigate whether task-related changes in prefrontal recruitment measured by fNIRS are affected by individual differences in people post-stroke. The first hypothesis was that during typical walking, people with poor mobility/motor function would exhibit higher prefrontal recruitment, consistent with compensatory over-recruitment. The second hypothesis was that during dual-task walking (with an added cognitive task), people with poor cognitive function would exhibit a lack of task-appropriate prefrontal recruitment (i.e., ceiling effect) and worse dual-task performance. Exploratory analyses were also conducted to examine the extent to which task-related differences in prefrontal recruitment explained task performance.

## Materials and Methods

### Participants

Thirty-three adults with chronic post-stroke hemiparesis and moderate to severe walking deficits were enrolled. The inclusion criteria for the study included age >21 years; at least 6 months post-stroke; medically stable; able to follow 3-stage commands; ability to walk without support from another person; 10-meter walking speed ≤0.8 m/s (Perry et al., 1995) and Fugl-Meyer lower extremity (FMA-LE) score <30 to ensure that the participants had substantial motor deficits (Fugl-Meyer et al., 1975). These clinical evaluations were conducted by a licensed physical therapist who also confirmed the presence of a hemiparetic walking deficit. Some participants used an ankle/foot orthosis and/or a cane if needed to safely complete the walking assessments (see Table 1). Stroke side and lesion location for each participant was determined from medical records, and the location was broadly categorized as: anterior cerebral artery (ACA) territory, middle cerebral artery (MCA) territory, basal ganglia (BG), and/or pons (see Table 1).

**Table 1:** Mean demographic and clinical data.

Age (years)	59.6 ± 9.7
Gender (Male/Female)	22/11
Affected Hemisphere (Left/Right)	16/17
Chronicity (months)	19.2 ± 10.4
10MWT (m/s)	0.6 ± 0.2
Fugl-Meyer LE score (out of 34)	24.7 ± 4.4
DGI (out of 24)	13.6 ± 3.5
MMSE (out of 30)	26.6 ± 3.1
ABC Scale (%)	59.2 ± 19.6
Lesion Location (ACA/MCA/BG&IC/Pons)	4/10/14/5
Assistive Device (AD) only	2
Ankle-foot Orthosis (AFO) only	6
AD + AFO	4

*Abbreviations: ABC, Activities-Specific Balance Confidence Scale; DGI, Dynamic Gait Index; Fugl-Meyer LE, Fugl-Meyer Lower Extremity Score; MMSE, Mini-Mental State Examination; 10MWT, 10-Meter Walk Test; ACA, Anterior Cerebral Artery Territory; MCA, Middle Cerebral Artery Territory; BG, Basal Ganglia; IC, Internal Capsule; AD, Assistive Device; AFO, Ankle-Foot Orthosis. The errors denote standard deviation*.

Exclusion criteria included Mini-Mental State Examination (MMSE) score <21 to exclude individuals with moderate to severe cognitive impairments from the study (Folstein et al., 1975); uncontrolled hypertension; lower extremity pain that would interfere with walking; severe obesity (body mass index >40); cardiovascular disease such as congestive heart failure, significant valvular disease, history of cardiac arrest, presence of an implantable defibrillator, uncontrolled angina; history of myocardial infarction or heart surgery in the prior year; lung disease requiring use of corticosteroids or supplemental oxygen; renal disease requiring dialysis; significant visual and/or vestibular impairment impacting safe mobility; lower motor neuron injury; bone fracture or joint replacement in the prior 6 months; diagnosis of a terminal illness. The study procedures were approved by the local Institutional Review Board and all participants provided written informed consent at the time of enrollment.

### Protocol and Equipment

All assessments were conducted at a research laboratory located in an outpatient hospital setting. Three tasks were assessed for this study: typical walking (*Walk*), serial-7 subtraction (*Serial7*), and combined typical walking plus serial-7 subtraction (*Dual-Task*). The *Serial7* task was performed in a seated position. This facilitated consistency across participants when assessing single task cognitive performance and related prefrontal activity, since even standing balance can be cognitively demanding for some people after stroke. For both *Walk* and *Dual-Task*, participants walked at their preferred self-selected speed for 2–3 consecutive laps on an 18-m oval-shaped walking path. An instrumented walkway (GAITRite, CIR Systems, PA, USA) was located on one side of the path to measure spatiotemporal gait data. For both *Serial7* and *Dual-Task*, participants were asked to continuously subtract by seven beginning from a randomly assigned number between 91 and 99 (Hayman, 1942; Williams et al., 1996). If the participant reached zero a new number was immediately assigned. The order of tasks was randomized. Participants were not given any special instructions pertaining to prioritization during *Dual-Task*. A small number of participants had expressive aphasia with consequent difficulty verbalizing their responses. These individuals were instructed to perform the serial-7 subtraction task silently to minimize the confounding effect.

fNIRS (Niro 200NX, Hamamatsu Photonics, Japan) was used to measure prefrontal recruitment from the anterior prefrontal cortex (Brodmann Area 10) during all tasks. fNIRS estimates neuronal activity in underlying tissue by calculating hemodynamic changes due to neurovascular coupling (Leff et al., 2011; Perrey, 2014). A diode emitted infrared light at continuous wavelengths of 735 nm and 810 nm. Changes in prefrontal oxygenated (O_2_Hb) and deoxygenated (HHb) hemoglobin concentration were estimated with the modified Beer-Lambert Law. Change in O_2_Hb concentration was used as the primary outcome measure of prefrontal recruitment because this measure has been consistently reported to be sensitive to walking-related changes in cortical activity (Miyai et al., 2001; Harada et al., 2009; Maidan et al., 2015).

For each channel (left and right side), a rubber probe holder was used to set optode spacing at 3 cm and to block ambient light. The optodes were secured to the forehead over the left and right anterior prefrontal cortices by double-sided adhesive. The optodes were placed high on the forehead to avoid the temporalis muscle and sufficiently lateral from the midline to avoid the superior sagittal sinus (Al-Rawi and Kirkpatrick, 2006; Tisdall et al., 2009). We have successfully implemented this procedure in our prior published work (Clark et al., 2014; Hawkins et al., 2018). To further minimize movement artifact in the signal, optodes and wires were secured by a fabric headband and the wires were also secured to the upper back. Prior to beginning each walking task, participants rested quietly in a standing position for approximately 1 min to provide a baseline level of prefrontal activity. Participants were not told exactly when the walking task would begin to prevent an anticipatory increase in prefrontal recruitment.

### Clinical Assessments

Preferred walking speed was measured with the 10-Meter Walk Test (10MWT). Cognitive function was measured with Mini-Mental State Examination (MMSE; Folstein et al., 1975). Balance confidence was measured by self-report with the Activities-specific Balance Confidence (ABC) Scale (Powell and Myers, 1995). Gait and balance function were measured with the Dynamic Gait Index (DGI; Jonsdottir and Cattaneo, 2007). Lower extremity sensorimotor impairment was measured with the lower extremity Fugl-Meyer Assessment (FMA-LE; Fugl-Meyer et al., 1975).

## Data Analysis

Prefrontal O_2_Hb data were analyzed with custom programs created with Matlab version R2015a (Mathworks, Natick, MA, USA). Data were sampled at 2 Hz and saved directly to a memory card in the data acquisition unit, and later downloaded to a computer for analysis. All data were inspected for signal artifact using procedures that were based on the recommendations of Cooper et al. (2012). Artifacts in the O_2_Hb signal were defined as amplitude offset exceeding 1 μM within a 2-s period, and/or a 2-s sliding window standard deviation that exceeded 3 standard deviations of the original full signal. All automatically detected artifacts were visually confirmed by a trained team member. The occurrence of artifacts was relatively infrequent (less than one per trial, on average) and transient. Artifacts were removed and replaced with linear interpolation to the surrounding data points.

Resting baseline prefrontal activity was quantified during the final 10 s of the rest period that preceded each task. For the active period, prefrontal activity was measured over a 30-s period that began 7 s after task onset in order to allow for cerebral blood flow changes to stabilize. This is based on prior work on neurovascular coupling, and is consistent with the recommendations for fNIRS measurement of peak task-related hemodynamic response which occurs approximately 6 s after the onset of neuronal activity (Cui et al., 2010; Tong and Frederick, 2010; Vitorio et al., 2017; Herold et al., 2018). The primary fNIRS outcome measure was the change in oxygenated hemoglobin concentration (ΔO_2_Hb) between the resting baseline period and active period within each task, calculated using the following equation: Prefrontal ΔO_2_Hb = Active O_2_Hb − Resting O_2_Hb.

Similarly, the change in deoxygenated hemoglobin concentration (ΔHHb) between the resting baseline period and active period within each task was calculated using the following equation: Prefrontal ΔHHb = Active HHb − Resting HHb.

## Statistical Analysis

Statistical analysis was conducted using JMP software (JMP^®^ 11. SAS Institute Inc., Cary, NC, USA). For all analyses, statistical significance level was set at alpha < 0.05. Paired *t*-tests were conducted to compare the magnitude of left and right prefrontal ΔO_2_Hb to examine whether the data were affected by the laterality of the cortical recording site. Pearson’s correlation coefficient was used to examine the consistency of prefrontal ΔO_2_Hb measured from the left and right prefrontal cortical recording sites.

### Confirmation of Dual-Task Cost

Paired *t*-tests were conducted to compare walking speed, stride length, and step width between *Walk* and *Dual-Task*, and to also compare cognitive performance between *Serial7* and *Dual-Task*. A one-way repeated measures analysis of variance (ANOVA) model was used to compare prefrontal ΔO_2_Hb across the three tasks. The assumption of sphericity for the ANOVA model was tested by Mauchly’s test ($\chi_{\left(2\right)}^{2}$ = 1.96, *p* = 0.37). Paired *t*-tests were conducted *post hoc* to compare prefrontal ΔO_2_Hb between *Dual-Task* and *Walk*, and between *Dual-Task* and *Serial7*. Separate paired *t*-tests were conducted to compare prefrontal ΔHHb between *Dual-Task* and *Walk*, and between *Dual-Task* and *Serial7*.

### Predictors of Task-Related Prefrontal ΔO_2_Hb and Task Performance

A stepwise mixed-model regression analysis was conducted to identify the variables that significantly predicted the magnitude of prefrontal ΔO_2_Hb during each task. The variables entered in the model included age, sex, stroke region, injured hemisphere, stroke chronicity, and performance on clinical assessments including 10MWT, ABC Scale, DGI, FMA-LE, and MMSE. All assumptions for multiple regression models were met. For each task, the criteria for entering the predictor variable into the regression model was set at *p* = 0.10 and exiting the model was set at *p* = 0.15. Based on the identified predictor(s), subgroups were formed (lower and higher functioning) and *t*-test used to compare prefrontal ΔO_2_Hb and task performance.

### Association Between Prefrontal ΔO_2_Hb and Task Performance

Pearson’s correlation coefficient was used to explore the associations between dual-task cost of prefrontal recruitment denoted as ΔO_2_Hb_cost_ (calculated as dual-task − single task) and dual-task cost of performance (calculated as dual-task − single task). Dual-task costs were calculated for both cognitive performance (serial-7 subtraction response rate) and walking performance (speed, stride length, and step width).

## Results

### Group Characteristics and Confirmation of Dual-Task Cost

Demographic and clinical data for all participants are presented in Table 1.

**Figure 1:**
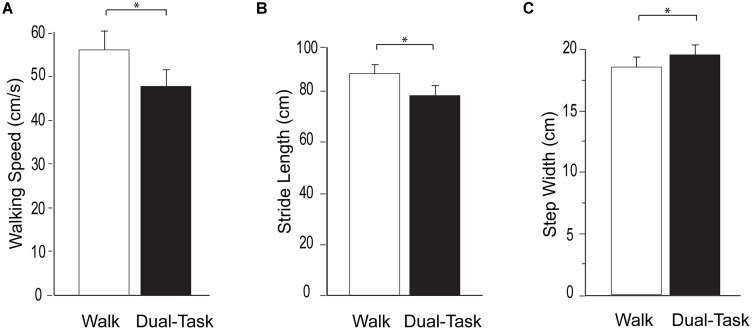
Group average data for the measurements of walking performance during typical walking (Walk) shown in white and dual-task walking (Dual-Task) shown in black. From left to right the graphs show walking speed **(A)**, stride length **(B)**, and step width **(C)**. The error bars denote the standard error. *p < 0.05.

Walking task performance is presented in Figure 1. Consistent with prior studies, a substantial dual-task cost was observed. Compared to *Walk, Dual-Task* walking speed was significantly slower (*p* < 0.0001), stride length was significantly shorter (*p* < 0.0001), and step width was significantly wider (*p* < 0.001). Likewise, compared to *Serial7*, cognitive performance deteriorated significantly during *Dual-Task* (0.13 ± 0.09 vs. 0.11 ± 0.08 responses/s; *p* = 0.01).

**Figure 2:**
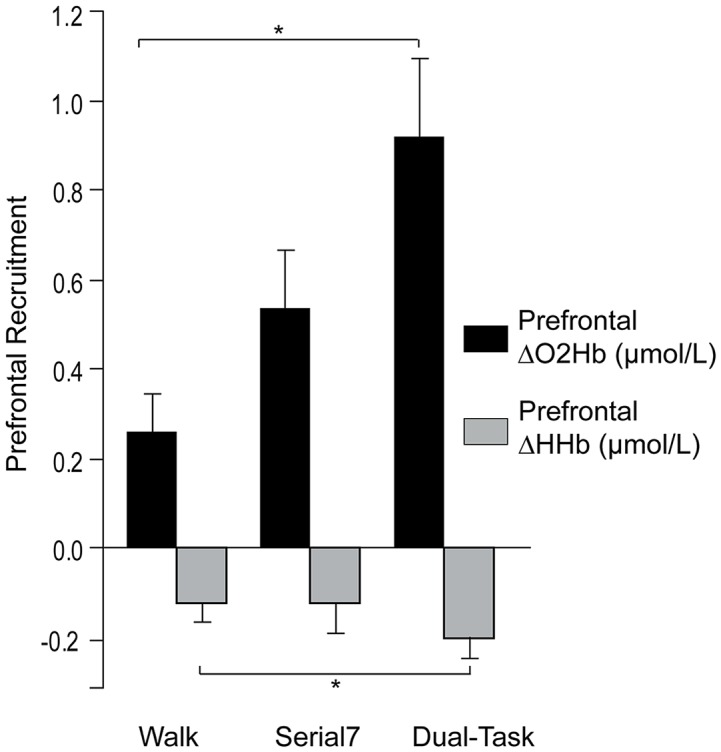
Group average data for the change in prefrontal oxygenated (ΔO_2_Hb) and deoxygenated (ΔHHb) hemoglobin concentration from resting period to the active period during single and dual-tasks. From left to right the graphs show ΔO_2_Hb (in black) and ΔHHb (in light gray) during typical walking (Walk), serial-7 single task (Serial7), and dual-task walking (Dual-Task). The error bars denote the standard error. *p < 0.05.

Consistent with prior reports, no effect of laterality on prefrontal ΔO_2_Hb was observed for the single and dual-tasks (Mirelman et al., 2014; Nieuwhof et al., 2016). The group mean ΔO_2_Hb was not significantly different between the left and right prefrontal cortex for *Serial7* (*p* = 0.20), *Walk* (*p* = 0.56), or *Dual-Task* (*p* = 0.76). Furthermore, ΔO_2_Hb was strongly correlated for the left and right prefrontal cortex for *Serial7* (*r* = 0.87, *p* < 0.0001), *Walk* (*r* = 0.79, *p* < 0.0001), and *Dual-Task* (*r* = 0.91, *p* < 0.0001). Therefore, prefrontal ΔO_2_Hb data from both hemispheres were averaged within each participant prior to all subsequent analyses. The magnitude of prefrontal ΔO_2_Hb varied across tasks (*p* < 0.01; Figure 2). *Post hoc* analysis revealed that prefrontal ΔO_2_Hb during *Dual-Task* was greater than *Walk* (*p* = 0.001; *d* = 0.79) and *Serial7* (trend with *p* = 0.06; *d* = 0.44). In agreement, prefrontal ΔHHb was more negative during *Dual-Task* than *Walk* (*p* = 0.03) and *Serial7* (trend with *p* = 0.11; Figure 2).

### Predictors of Task-Related Prefrontal ΔO_2_Hb and Task Performance

Examination of collinearity between the predictors revealed that DGI was significantly correlated with the 10MWT (*r* = 0.64, *p* < 0.0001), FMA-LE (*r* = 0.45, *p* = 0.006), and MMSE (*r* = 0.46, *p* = 0.005) scores. 10MWT was significantly correlated with ABC Scale (*r* = 0.45, *p* = 0.006), and FMA-LE (*r* = 0.41, *p* = 0.01) scores. FMA-LE was significantly correlated with chronicity of stroke (*r* = 0.36, *p* = 0.04). DGI was excluded from the stepwise mixed-model regression analysis to ensure that collinearity between the predictor variables did not influence the study findings. The mean variance inflation factor (VIF) was 1.85 before and 1.41 after the removal of DGI which is well within the recommended limits (Hair et al., 1995; Kutner et al., 2005; Kennedy, 2008).

### Prefrontal ΔO_2_Hb During *Walk*

**Figure 3:**
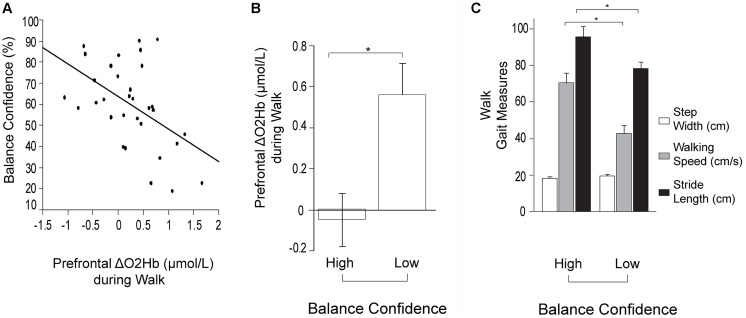
Prefrontal oxygenated hemoglobin (ΔO_2_Hb) concentration and the measurements of walking performance during typical walking (Walk). The error bars denote the standard error. *p < 0.05. **(A)** Association between prefrontal recruitment (ΔO_2_Hb) during typical walking (Walk) and balance confidence (%) measured by the Activities-specific Balance Confidence (ABC) Scale. **(B)** Comparison of prefrontal recruitment (ΔO_2_Hb) between the High (left) and Low (right) Balance Confidence groups during typical walking (Walk). **(C)** Comparison of walking performance between the High (left) and Low (right) Balance Confidence groups during typical walking (Walk). The graphs show step width in white, walking speed in light gray, and stride length in black.

For the stepwise regression model assessing predictors of prefrontal ΔO_2_Hb during *Walk* (*R*^2^ = 0.33; Table 2), lower self-reported balance confidence measured by the ABC Scale (*p* = 0.003) and lower FMA-LE score (trend with *p* = 0.09) were associated with greater prefrontal ΔO_2_Hb. The bivariate correlation between balance confidence and prefrontal ΔO_2_Hb was *r* = 0.48, *p* = 0.004 (Figure 3A). Based on this finding, further investigation was conducted by subdividing participants into Low and High Balance Confidence groups based on the median of the ABC Scale scores. Participants scoring ≤58.75% were placed in the Low Balance Confidence group. Demographic and clinical data for the balance confidence subgroups are presented in Table 3. ABC Scale scores were confirmed to be significantly lower in the Low Balance Confidence group (44.5% ± 13.3 vs. 75.0% ± 10.9, *p* < 0.001). The Low Balance Confidence group exhibited significantly higher prefrontal ΔO_2_Hb (*p* = 0.002; Figure 3B), slower walking speed (*p* < 0.001), and shorter stride length (*p* = 0.008) during *Walk* (Figure 3C).

**Table 2:** Stepwise regression table showing the predictors of task-related prefrontal ΔO_2_Hb.

Variables	Tasks								
	Walk			Serial7			Dual-Task		
	Estimate	*R*^2^	*p*-value	Estimate	*R*^2^	*p*-value	Estimate	*R*^2^	*p*-value
Age	-	-	0.63	-	-	0.86	-	-	0.62
Sex	-	-	0.99	-	-	0.36	-	-	0.81
Stroke Region (BG-Pons and MCA and ACA)	-	-	0.30	-	-	0.14	-	-	0.59
Stroke Region (Pons-MCA and ACA)	-	-	0.68	-	-	0.87	-	-	0.59
Stroke Region (MCA)	-	-	0.92	-	-	0.59	-	-	0.89
Injured Hemisphere (Left/Right)	-	-	0.80	-	-	0.53	-	-	0.70
Chronicity	-	-	0.92	-	-	0.97	-	-	0.58
10-Meter Walking Speed	-	-	0.46	-	-	0.99	-	-	0.85
Balance Confidence (ABC Scale)	−0.016	0.25	0.003*	-	-	0.31	-	-	0.26
Fugl-Meyer lower extremity score	−0.040	0.33	0.09	-	-	0.20	−0.069	0.36	0.05
Mini-Mental State Examination	-	-	0.62	−0.111	0.18	0.02*	0.188	0.25	0.002*

Abbreviations: ABC, Activities-Specific Balance Confidence Scale; ACA, Anterior Cerebral Artery; BG, Basal Ganglia; MCA, Middle Cerebral Artery. Predictors of change in task-related prefrontal oxygenated hemoglobin (ΔO_2_Hb) concentration based on the findings of the stepwise mixed-model regression analysis. *p < 0.05.

**Table 3:** Demographics and clinical assessments for balance confidence subgroups.

	Balance confidence subgroups	
Clinical assessments	Low (*n* = 17)	High (*n* = 16)
Age (years)	57.8 ± 10.6	61.6 ± 8.5
Chronicity (months)	19.6 ± 10.9	18.8 ± 10.1
*10MWT (m/s)	0.4 ± 0.2	0.7 ± 0.2
Fugl-Meyer LE score (out of 34)	23.9 ± 5.2	25.6 ± 3.1
DGI (out of 24)	12.9 ± 2.5	14.4 ± 4.3
MMSE (out of 30)	26.4 ± 3.2	26.8 ± 3.1
*ABC Scale (%)	44.4 ± 13.3	75.0 ± 10.8

Abbreviations: ABC, Activities-Specific Balance Confidence Scale; DGI, Dynamic Gait Index; Fugl-Meyer LE, Fugl-Meyer Lower Extremity Score; MMSE, Mini-Mental State Examination; 10MWT, 10-Meter Walk Test. The errors denote standard deviation. *p < 0.05.

### Prefrontal ΔO_2_Hb During *Serial7* and *Dual-Task*

For the stepwise regression model assessing predictors of prefrontal ΔO_2_Hb during *Serial7*, lower MMSE score was associated with greater prefrontal ΔO_2_Hb (*R*^2^ = 0.18, *p* = 0.02). For the stepwise regression model assessing predictors of ΔO_2_Hb during the *Dual-Task* (*R*^2^ = 0.36; Table 2), higher MMSE scores (*p* = 0.002) and lower FMA-LE scores (trend with *p* = 0.05) were associated with greater prefrontal ΔO_2_Hb.

**Figure 4:**
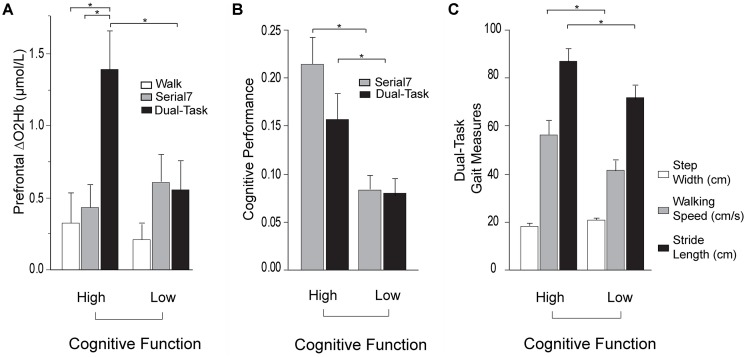
Prefrontal oxygenated hemoglobin (ΔO_2_Hb) concentration, cognitive performance, and the measurements of walking performance in High (left) and Low (right) Cognitive Function groups during single and dual-tasks. The error bars denote the standard error. *p < 0.05. **(A)** Comparison of prefrontal recruitment (ΔO_2_Hb) between the High (left) and Low (right) Cognitive Function groups during typical walking (Walk) in white, serial-7 single task (Serial7) in light gray, and dual-task walking (Dual-Task) in black. **(B)** Comparison of serial-7 response rate between the High (left) and Low (right) Cognitive Function groups for serial-7 single task (Serial7) in light gray and dual-task walking (Dual-Task) in black. **(C)** Comparison of walking performance between the High (left) and Low (right) Cognitive Function groups during dual-task walking (Dual-Task). The graphs show step width in white, walking speed in light gray, and stride length in black.

Based on this finding, further investigation was conducted by subdividing participants into Low and High Cognitive Function groups based on the median of the MMSE scores. Participants scoring ≤27 were placed in the Low Cognitive Function group. Demographic and clinical data for the cognitive subgroups are presented in Table 4. MMSE scores were confirmed to be significantly lower in the Low Cognitive Function group (24.7 ± 3.0 vs. 29.2 ± 0.8, *p* < 0.001). Cognitive performance was also significantly worse in the Low Cognitive Function group compared to the High group for *Serial7* (0.08 ± 0.06 vs. 0.21 ± 0.09 responses/s, *p* < 0.001) and *Dual-Task* (0.07 ± 0.06 vs. 0.15 ± 0.09 responses/s, *p* = 0.01; Figure 4B). Similarly, walking performance during *Dual-Task* (Figure 4C) was worse in the Low Cognitive Function group, as exhibited by slower walking speed (*p* = 0.03), shorter stride length (*p* = 0.02), and a trend for wider step width (*p* = 0.10).

**Table 4:** Demographics and clinical assessments for cognitive function subgroups.

	Cognitive function subgroups	
Clinical assessments	Low (*n* = 19)	High (*n* = 14)
Age (years)	57.8 ± 8.8	62.1 ± 10.7
Chronicity (months)	20.0 ± 10.8	17.9 ± 10.0
10MWT (m/s)	0.5 ± 0.2	0.6 ± 0.2
Fugl-Meyer LE score (out of 34)	23.8 ± 4.2	25.9 ± 4.4
*DGI (out of 24)	12.4 ± 3.3	15.3 ± 3.2
*MMSE (out of 30)	24.7 ± 2.9	29.2 ± 0.8
ABC Scale (%)	59.5 ± 17.8	58.9 ± 22.4

Abbreviations: ABC, Activities-Specific Balance Confidence Scale; DGI, Dynamic Gait Index; Fugl-Meyer LE, Fugl-Meyer Lower Extremity Score; MMSE, Mini-Mental State Examination; 10MWT, 10-Meter Walk Test. The errors denote standard deviation. *p < 0.05.

For *Dual-Task*, prefrontal ΔO_2_Hb was significantly higher in the High Cognitive Function group compared to the Low Cognitive Function group (*p* = 0.01; Figure 4A). The High Cognitive Function group exhibited a task-appropriate increase in prefrontal recruitment during dual-tasking, as demonstrated by significantly higher prefrontal ΔO_2_Hb during *Dual-Task* compared to both *Serial7* (*p* = 0.006) and *Walk* (*p* = 0.0008). In contrast, the Low Cognitive Function group exhibited similar prefrontal ΔO_2_Hb during *Dual-Task* compared to *Serial7* (*p* = 0.73), and only a trend for higher prefrontal ΔO_2_Hb during *Dual-Task* compared to *Walk* (*p* = 0.10).

### Association Between Prefrontal ΔO_2_Hb and Task Performance

**Figure 5:**
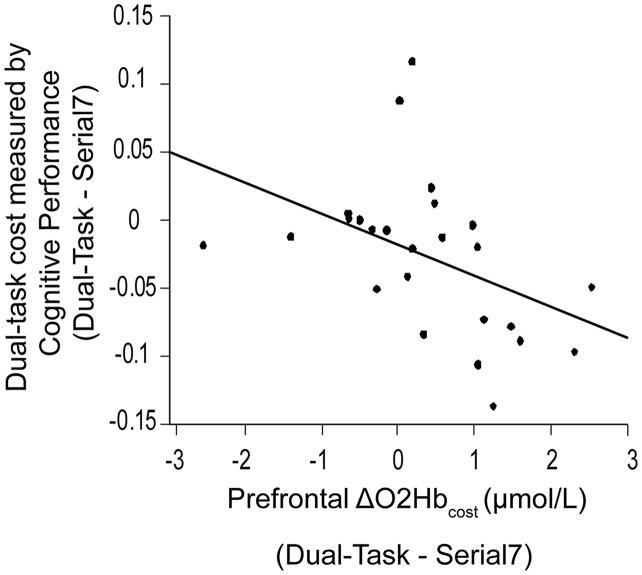
Association between dual-task cost measured by cognitive performance, and the change in prefrontal oxygenated hemoglobin (ΔO_2_Hb_cost_) concentration from serial-7 single task (Serial7) to dual-task walking (Dual-Task). Higher task-based increase in prefrontal recruitment (dual-task minus single task) is associated with worse dual-task cost measured by cognitive performance (i.e., a greater drop in serial-7 response rate).

**Figure 6:**
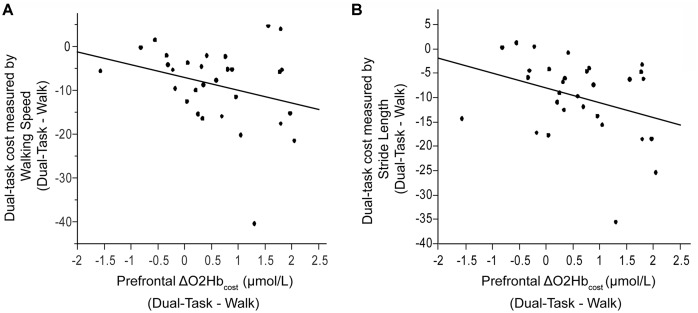
Association between dual-task cost measured by walking speed (Panel **A**) and stride length (Panel **B**), and the change in prefrontal oxygenated hemoglobin (ΔO_2_Hb_cost_) concentration from typical walking (Walk) to dual-task walking (Dual-Task). Higher task-based increase in prefrontal recruitment (dual-task minus single task) is associated with worse dual-task cost measured by walking speed and stride length.

Also examined was the extent to which dual-task costs of prefrontal recruitment were associated with dual-task costs of cognitive performance and walking performance. For cognitive performance (*Dual-Task—Serial7*), higher prefrontal ΔO_2_Hb_cost_ was associated with worse serial-7 subtraction cost (*r* = 0.43, *p* = 0.02; Figure 5). For walking performance (*Dual-Task—Walk*), higher prefrontal ΔO_2_Hb_cost_ showed a trend for an association with slowing of walking speed (*r* = 0.28, *p* = 0.11; Figure 6A), as well as with shorter stride length (*r* = 0.33, *p* = 0.06; Figure 6B).

## Discussion

The objective of this study was to investigate whether task-related changes in prefrontal cortical recruitment measured by fNIRS are affected by individual differences in people post-stroke. The primary hypotheses were that people with poor mobility function would exhibit higher prefrontal recruitment (i.e., compensatory over-recruitment) during typical walking, and people with poor cognitive function would exhibit lower prefrontal recruitment (i.e., recruitment ceiling effect) during dual-task walking.

### Typical Walking (*Walk*): Prefrontal Recruitment and Task Performance

Based on the results from the stepwise regression model, greater prefrontal recruitment during *Walk* was strongly associated with lower self-reported balance confidence, as measured with the ABC Scale (Table 2). There is strong evidence from prior work that supports the important role of balance confidence (and related measures of mobility self-efficacy) as an independent predictor of walking-related activity and participation, even after accounting for deficits in physical function (Robinson et al., 2011; Danks et al., 2016). Paretic leg motor dysfunction (i.e., poorer FMA-LE scores) also showed a trend for predicting higher levels of prefrontal recruitment during *Walk*, as previously reported (Hawkins et al., 2018). One explanation for this finding is that individuals with more severe mobility/motor deficits after stroke exhibit compensatory recruitment of executive control resources to counter the loss of automatic/healthy control mechanisms. Indeed the “cautious” gait behaviors (slower walking speed and shorter stride length; Figure 3C) in the subgroup with lower balance confidence is consistent with a more cognitively demanding control strategy. These findings also build upon prior evidence from people post-stroke and other mobility compromised populations. For instance, prefrontal over-recruitment during walking has previously been reported in people post-stroke compared to healthy adults without neurologic deficits (Mihara et al., 2007; Hawkins et al., 2018), as well as in other clinical populations including people with ataxia (Caliandro et al., 2012), Parkinson’s disease (Maidan et al., 2016a,b), and Multiple Sclerosis (Hernandez et al., 2016).

### *Serial7* and *Dual-Task*: Prefrontal Recruitment and Task Performance

Prefrontal recruitment during *Dual-Task* was higher than either of the single tasks (i.e., *Walk* and *Serial7*), which is consistent with the greater cognitive demands of dual-tasking (Figure 2). For *Serial7*, higher MMSE score was found to be a predictor of lower prefrontal recruitment. This finding suggests that when people perform a relatively low-demand task (e.g., single task compared to a dual-tasking condition), those with better cognitive function exhibit more efficient prefrontal recruitment. That is, fewer resources are needed to accomplish the task. This is generally consistent with the aforementioned result from typical walking (people with better mobility function exhibited less prefrontal recruitment), although the mechanistic reasoning might differ as explained in the “*Introduction”* section.

For *Dual-Task*, better MMSE score and worse FMA-LE score were associated with greater prefrontal recruitment (Table 2). Notably, the direction of the MMSE association is reversed from what was observed in *Serial7*. As discussed above, neural efficiency (where less recruitment is “better”) may have been the dominant factor driving the association in the relatively less demanding *Serial7* task. In contrast, the more demanding *Dual-Task* condition may cause some individuals to reach a ceiling of resource recruitment. In this case, higher recruitment can be considered “better” if due to greater availability of cognitive resource reserves. The recruitment ceiling might be the dominant factor driving the association for *Dual-Task*. To further investigate these data, participants were divided into two subgroups based on the median of MMSE scores. Within each subgroup, prefrontal recruitment was compared for the *Serial7* vs. *Dual-Task* condition. The Low Cognitive Function subgroup did not exhibit a significant change in prefrontal recruitment between the conditions (Figure 4A), which further suggests a ceiling effect such that both tasks were performed at or near the maximal prefrontal recruitment capability. This apparent ceiling effect might indicate that the Low Cognitive Function group did not have available resources to recruit for optimal performance of either *Serial7* or *Dual-Task*, which would help to explain the poor performance of this group on both tasks. In marked contrast, the High Cognitive Function group exhibited a substantial increase in prefrontal recruitment during *Dual-Task* relative to *Serial7*, which suggests that *Serial7* prefrontal recruitment was well below the recruitment ceiling (Figure 4A). This is consistent with more efficient brain processing and relative ease of performance during *Serial7* and is in agreement with the much better cognitive performance demonstrated by this group (Figure 4B). Cumulatively, these data are consistent with the hypothesis that during *Dual-Task*, people with poor cognitive function exhibit a lack of task-appropriate prefrontal recruitment that might be due to a recruitment ceiling effect.

It is unclear whether the greatly increased recruitment during *Dual-Task* compared to either of the single task conditions in the High Cognitive Function group is an indicator of good or poor neural control. As noted in prior studies, increased brain recruitment might support better performance, or a lack of performance ability might elicit increased brain recruitment as a compensation (Cabeza, 2002; Cabeza et al., 2002; Reuter-Lorenz and Cappell, 2008; Schneider-Garces et al., 2010; Holtzer et al., 2011; Clark et al., 2014; Hawkins et al., 2018). For the single task conditions, better performance was accompanied by lower levels of prefrontal recruitment consistent with efficient processing and/or absence of compensatory recruitment. A large increase in brain recruitment for *Dual-Task* might reflect poor efficiency of neural circuits and/or difficulty performing the task, which leads to additional recruitment. Indeed, further analysis shows that individuals with a greater increase in prefrontal recruitment (for *Dual-Task* relative to *Serial7*) also exhibit a greater decrement in serial-7 response rate (i.e., greater dual-task cost; Figure 5). Likewise, a trend between higher prefrontal recruitment and greater dual-task cost during walking was observed. The increased prefrontal recruitment during dual-tasking in the High Cognitive Function group might instead reflect the positive use of available resources to support task performance. While the notion of a higher ceiling in this subgroup is certainly supported, there is a lack of support that this higher ceiling led to benefits in task performance. Within the High Cognitive Function group, serial-7 performance (Figure 4B) and walking speed (Figure 4C) dropped significantly from single to dual-task despite the large increase in prefrontal recruitment. While it is possible that the heightened prefrontal recruitment prevented an even more precipitous performance decline, this assertion cannot be fully tested with the present data. Furthermore, the Low Cognitive Function group had no drop in serial-7 performance from single to dual-task (Figure 4B). This was despite also lacking the potential compensatory benefit of additional prefrontal recruitment. However, it should be acknowledged that the Low Cognitive Function group already had extremely low response rates for *Serial7* (less than one correct response per 10 s; approximately 70% lower than the High Cognitive Function group). They also walked at a slower walking speed during *Walk*, which slowed even more for *Dual-Task*. Therefore, the performance of this group was already very poor and there was little room to drop further. Furthermore, the absolute task demand for this group was lower, given that walking speed, stride length, and rate of serial-7subtraction response were significantly lower. These factors would have made the task easier compared to if walking speed and rate of serial-7 computation rate was matched between the Low and High Cognitive Function subgroups. More detailed insights could be gained by standardizing dual-task difficulty such as by asking individuals to walk at a predetermined walking speed (e.g., controlled by a treadmill) and standardizing the rate of computational items (e.g., a fixed number of serial-7 subtractions per minute).

The findings of this study have important implications for real-world mobility function and participation. These findings build upon existing evidence showing that adults with neurologic impairments have a poorer capability for multitasking and appear to reach a ceiling in resource recruitment more easily than healthy adults (O’Shea et al., 2002). Consequently, individuals with neurologic impairments may experience a greater decline in performance when performing concurrent cognitive and motor tasks, such as more severe gait deficits and postural instability during walking (Morris et al., 1996; Hollman et al., 2006). This may increase the susceptibility to falls (Lundin-Olsson et al., 1997; Hyndman and Ashburn, 2004; Weerdesteyn et al., 2008; Nordin et al., 2010). Additionally, a broader implication of the reduced capacity to dual-task could be the inability to continue living independently (Oppewal and Hilgenkamp, 2017), and restricted participation in societal roles (Lord et al., 2004; D’Alisa et al., 2005; Rosen et al., 2005).

## Limitations and Future Directions

A methodological limitation of this study is that cortical activity was recorded from only a small region of cerebral cortex. Several other cerebral regions are also involved in the executive control of walking and should be examined by future studies (Hamacher et al., 2015; Metzger et al., 2017).

The findings of this study demonstrate that the interaction between task demands and individual characteristics play an important role in how prefrontal recruitment may be interpreted during walking tasks. This, in turn, can motivate the selection of intervention approaches to improve walking function. People with higher prefrontal recruitment during typical walking, interpreted as over-recruitment, may benefit from intervention approaches that aim to reduce the demand for executive control of walking. The present data suggests two potential targets; improving balance confidence and/or addressing impaired lower limb voluntary control. People with lower prefrontal recruitment during dual-task walking, interpreted as a recruitment ceiling, may benefit from intervention approaches that attempt to increase resource capacity. Possible approaches might include cognitive or dual-task training, perhaps with neuromodulatory adjuvants (e.g., pharmacological or non-invasive brain stimulation). Enhancing recovery of walking function by optimizing brain recruitment is an important area for future research investigations.

## Data Availability

The datasets generated for this study are available on request to the corresponding author.

## Ethics Statement

This study was carried out in accordance with the recommendations of the University of Florida Institutional Review Board with written informed consent from all subjects. All subjects gave written informed consent in accordance with the Declaration of Helsinki. The protocol was approved by the University of Florida Institutional Review Board and the North Florida/South Georgia Veterans Affairs Human Research Protection Program.

## Author Contributions

DC and EF designed the study. DC, SC, KH, DO and JS did the data collection. SC, DC, KH and JS did the data analysis. DC, EF, SC, KH, DO, JS, JD, DR, SW, EC and KB contributed towards the interpretation and preparation of the manuscript.

## Conflict of Interest Statement

The authors declare that the research was conducted in the absence of any commercial or financial relationships that could be construed as a potential conflict of interest.
